# First record of the cicada genus *Semia* Matsumura (Hemiptera, Cicadidae) from Vietnam, with the description of one new species and a key to species

**DOI:** 10.3897/zookeys.174.2242

**Published:** 2012-03-09

**Authors:** Hong-Thai Pham, Masami Hayashi, Jeng-Tze Yang

**Affiliations:** 1 Department of Insect Systematics, Institute of Ecology and Biological Resources (IEBR), 18 Hoang Quoc Viet St, Hanoi, Vietnam; 2 Department of Biology, Faculty of Education, Saitama University, Saitama 338-8570, Japan; 3 Department of Entomology, National Chung Hsing University, Taichung 402, Taiwan, R.O.C.

**Keywords:** New record genus, *Semia spinosa*, morphology, Cicadina, Auchenorrhyncha

## Abstract

The first record of the genus *Semia* Matsumura (Cicadidae: Cicadinae, Cicadini) from Vietnam is presented. One new species, *Semia spinosa* **sp. n.**, is described from southern Vietnam. Photos of the adult, illustrations of the male genitalia, a distribution map and biological data are provided. A key to the species of *Semia* based on the male adults is also given.

## Introduction

The cicada fauna of Vietnam has received little attention since the descriptions of Distant (1913a, b, 1917a, b). According to previous reports, 133 cicada species are known from Vietnam, representing 45 genera in all three subfamilies, Cicadinae, Cicadettinae and Tettigadinae (Lee (2008), Pham and Yang (2009, 2010) and Pham et al. (2010)). So far only two species of *Semia* have been described, *Semia watanabei* (Matsumura, 1907), the type species, from Taiwan and *Semia klapperichi* Jacobi, 1944, from Fukien Province, China. Here we describe a third species, *Semia spinosa* sp. n., from Dong Nai Province, southern Vietnam.

*Semia* is similar to *Leptosemia* Matsumura, 1917, *Terpnosia* Distant, 1892and *Euterpnosia* Matsumura, 1917, but has a dentate lateral margin to the pronotum (Fig. 2A), transverse male opercula that are nearly contiguous with each other (Fig. 2B) and lacks a tooth-like projection laterally on the male 4^th^ abdominal segment (Lee and Hayashi 2003).

## Materials and methods

Four males of the new species *Semia spinosa* sp. n., were collected from the Phu Ly, Ma Da-Vinh Cuu Nature reserve (NR), Vinh Cuu district, Dong Nai Province in southern Vietnam. The holotype and two paratypes are deposited in the Institute of Ecology and Biological Resources, Hanoi, Vietnam (IEBR), and one paratype in the Natural History Museum, London (BMNH).

**Figure 1.**
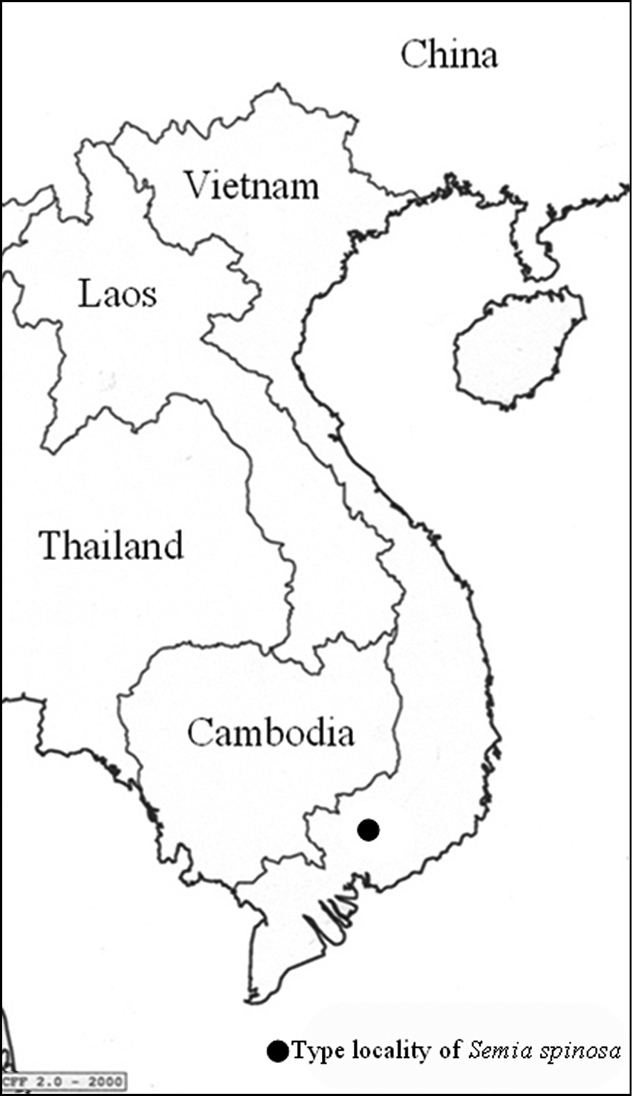
Type locality of *Semia spinosa* sp. n.; (see text for further details).

**Figure 2.**
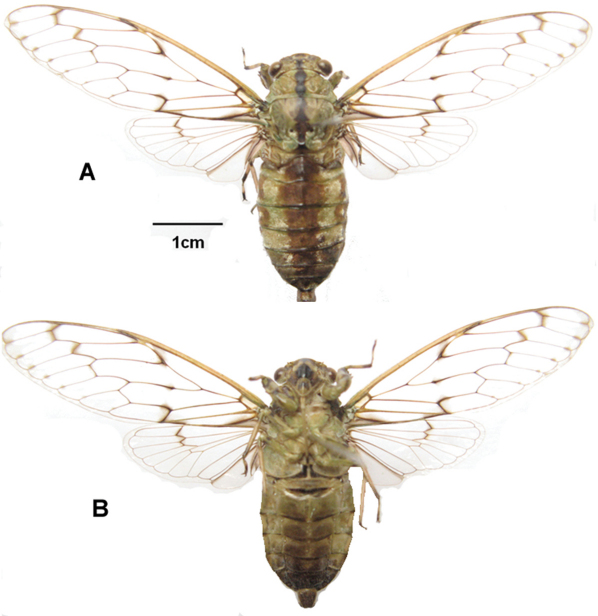
*Semia spinosa* sp. n. (male): **A** dorsal view **B** ventral view.

Nomenclature for family, subfamily and tribal classification follows that of Moulds (2005) and Lee (2008). Morphological terminology follows that of Moulds (2005). The male genitalia of the holotype were examined and photographed using a dissecting microscope (Leica MZ7 5). A distribution map (Fig. 1) produced by the software *CFF* 2.0 (Barbier and Rasmont 2000), and photos of habitus are provided (Fig. 2).

## Taxonomy

### Family Cicadidae Latrielle. Subfamily Cicadinae. Tribe Cicadini. Subtribe Cicadina

#### Genus Semia

Matsumura, 1917

http://species-id.net/wiki/Semia

Semia Matsumura, 1917: 195. Type species: *Leptopsaltria watanabei* Matsumura, 1907 (Formosa).

##### Diagnosis.

Head nearly as wide as or slightly narrower than base of mesonotum; inner area of pronotum generally concolorous to outer dilatation; male abdomen cylindrical, much longer than distance from head to cruciform elevation and slightly widest across 4th abdominal segment and wider than base of mesonotum; male tymbal cover very small and semicircular, mostly exposing tymbal in dorsal view; male 8th abdominal tergum mostly covered with white powder; ovipositor not protruding beyond abdominal segment 9; male operculum scale-like, roundish, and not extending beyond 2nd abdominal sternum; wings hyaline; 6th apical cell of forewing about as long as or longer than twice of 5th apical cell in median length. Based on Lee and Hayashi (2003).

##### Distribution.

China, Taiwan, Vietnam (Fig. 5).

##### Remarks.

 This genus is similar to *Leptosemia*, *Terpnosia* and *Euterpnosia* (see Introduction).

#### Semia spinosa sp. n.

urn:lsid:zoobank.org:act:B8A6B87E-9A4A-484C-90DE-4C4F7CFB2D8D

http://species-id.net/wiki/Semia_spinosa

Figs 2 3A–B 6C

##### Etymology.

The species name refers to morphological feature such as spinosa for the uncus spines

##### Material examined.

Holotype ♂: VIETNAM [VC.Ho.0650, Phu Ly, Ma Da-Vinh Cuu NR, Dong Nai Province, 4.viii.2008, light trap, 11°24'42.4"N, 107°06'19.5"E, 100–150m, leg Hoang Vu Tru] (IEBR).

Paratypes (3 ♂♂): 2 ♂♂: VIETNAM [ VC.Ho.0626, 0631, Phu Ly, Ma Da-Vinh Cuu NR, Dong Nai Province, 31.vii.2008, 100–150m, leg Hoang Vu Tru] (IEBR); 1 ♂: [VC.Ho.0765, Phu Ly, Ma Da-Vinh Cuu NR, Dong Nai Province, 2.viii.2008, light trap and netting, 100-150m, leg Ta Huy Thinh] (BMNH).

##### Description.

*Head* (Figs 2A, 2B, 7, 8): head pale yellowish-brown with following markings: broad median longitudinal band on frons and supra-antennal plate, dark brown; postclypeus dorsally with two oblique oval dark brown patches, in facial view upper half with transverse brown bands, lower half blackish brown; lower half of anteclypeus blackish brown, area between eye and antenna on gena, lorum and apex of rostrum, dark brown. Head including eyes as wide as mesonotum at base; rostrum reaching posterior coxae.

*Thorax* (Figs 2A, 2B): pale yellowish-green, longitudinal broad band on pronotum narrowed centrally, longitudinal broad band on mesonotum, spot between submedian and lateral sigillae, scutal depression, two spots on lateral margin of mesonotum, central area of cruciform elevation, second anepisternum, anepimeron and katepisternum, dark brown. Pronotal collar with a small dentate projection.

*Wings* (Fig. 2A): fore and hind wings hyaline, with veins brown or fuscous, and costal margin tawny; fore wings slightly tinged and spotted with infuscations on most veins.

*Legs* (2B): pale yellow with markings as follows: fore leg with femur, tibia, metatarsus and pretarsus blackish brown, primary spine of femur dark brown, secondary spine dark brown; mid leg with coxa and femur pale yellow, tibia pale brown, apex of femur black, apex and base of tibia black, metatarsus and pretarsus dark brown, mesotarsus pale yellow; hind leg, with femur pale yellow, apex of femur dark brown, tibia pale brown, base of tibia dark brown, tibial spur, tibial comb, and thumb of tibial comb dark brown.

*Abdomen* (Fig. 2B): pale greenish brown in dorsal view, with a longitudinal broad dark brown streak, tergites 3–7 with their lateral margins edged pale brown (Fig. 2A); pale brown in ventral view, anterior margin of sternites III - VI and sternites VII and VIII dark brown; epipleurites 3–6 lighter than sternites (Fig. 2B).

*Operculum* (Fig. 2B): pale yellow-green, short, transverse, and not reaching beyond anterior margin of sternite II.

**Figure 3.**
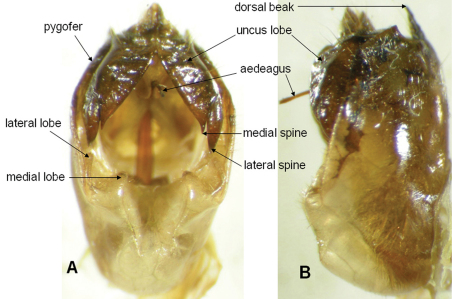
Male genital capsule of *Semia spinosa* sp. n.: **A** ventral view **B** lateral view.

*Male genitalia* (Figs 3A, B): Pygofer oblong in ventral view, lateral lobe of pygofer narrower than medial lobe, the latter triangular and prominent; dorsal beak acute and blackish brown; uncus brown, darker at apex of uncus lobes, the latter strongly divergent with two apical spines, medial spine shorter and acuter than lateral spine; Anal styles and anal tube dark brown. Aedeagus very slender.

*Measurements in mm:* (4 ♂♂): body length excluding wings: 27.1–29.0 (28.1); fore wing length: 31.0–32.6 (31.8); head width: 6.7–7.2 (7.0); pronotum width: 6.7–8.1 (7.4).

##### Biology.

 This species was collected by sweeping during the daytime and by light trapping at night in virgin rainforest at an altitude between 100 to150 meters.

##### Distribution.

Vietnam (Dong Nai Province).

##### Remarks.

**Figure 4.**
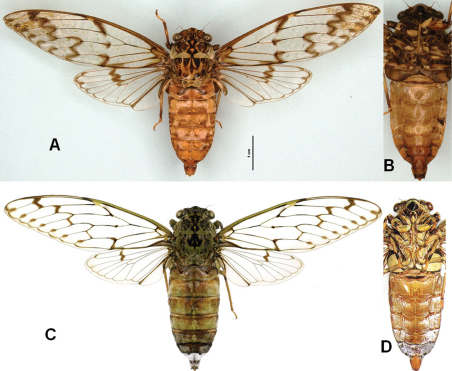
*Semia* species in dorsal view and ventral view (male): **A**, **B***Semia klapperichi*(photograph by Dirk Ahrens) **C, D** *Semia wantanabei* (after Chen 2007).

*Semia spinosa* is distinguishable from *Semia watanabei* and *Semia klapperichi* by the body size, which is shorter than 30mm (in male) in *Semia spinosa* and longer than 35mm (in male) in *Semia watanabei* and *Semia klapperichi*, and from *Semia klapperichi* it differs in the infuscations on the hind wings which lack spots along the ambient veins present in *Semia klapperichi* (see Figs 4A, C). The new species also differs in the structure of the uncus which has the lobes strongly divergent with acute apical spines (see Figs 6A–C).

**Figure 5.**
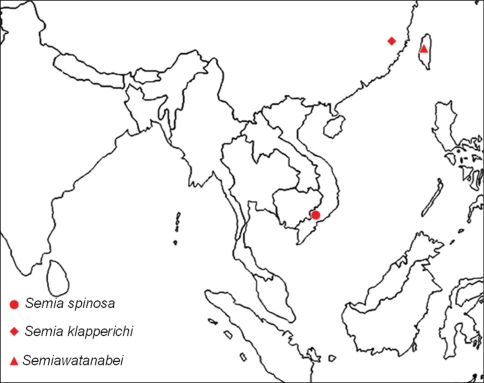
Distribution of the *Semina* species in the world.

**Figure 6.**
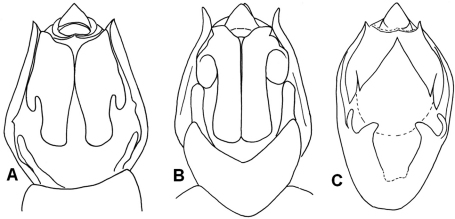
Male genital capsule of *Semia* species (ventral view): **A** *Semia klapperichi* **B** *Semia watanabei* (after Lee and Hayashi 2003) **C** *Semia spinosa* sp. n.

**Figure 7.**
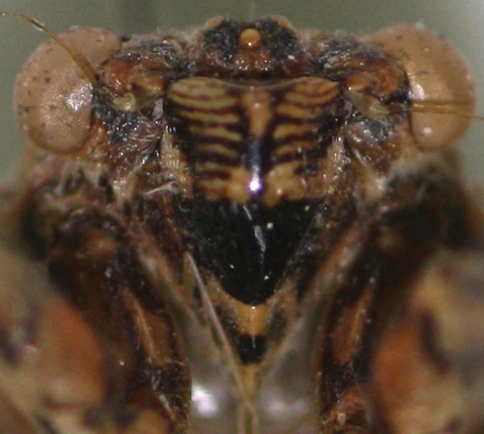
Postclypeus of *Semia spinosa* sp. n.

**Figure 8.**
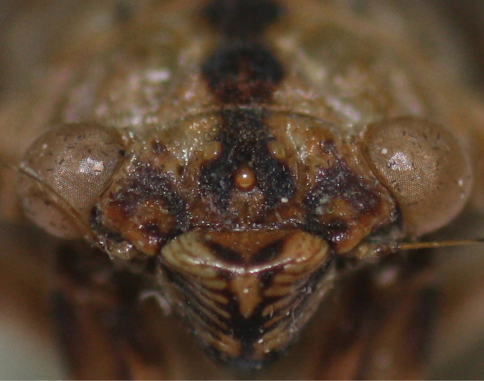
Dorsal part of the postclypeus of *Semia spinosa* sp. n.

### Key to the species of the genus Semia (males)

**Table:** 

1	Body length <30mm; abodomen with longitudinal broad brown band centrally (Fig. 2A); uncus lobes strongly divergent, each with two apical spines (Fig. 3A)	*Semia spinosa* sp. n.
–	Body length >35mm; abodomen without longitudinal broad brown band centrally; uncus lobes not or weakly divergent without two apical spines	2
2	Body length approximately 38mm; operculum with lateral margin not expanding beyond lateral margin of abdomen; tymbal cover very small, pale brown, with darker margin; tymbal mostly exposed in dorsal view; uncus lobes parallel (Fig. 6B)	*Semia watanabei*
–	Body length 40–45mm; operculum very wide, lateral margin expanding distinctly beyond lateral margin of abdomen; tymbal cover small slightly raised, brown without dark margin; tymbal slightly exposed in dorsal view; uncus lobes weakly divergent (Fig. 6A)	*Semia klapperichi*

## Supplementary Material

XML Treatment for Semia

XML Treatment for Semia spinosa
